# 

**Published:** 1917-07

**Authors:** 


CONTENTS.

*Page*

Fatigue Induced by Labour. By A. F. STANLEY KENT, M.A., D.Sc. Oxon. ... ... ... ... ... ... ... ... ... ... 47

Gunshot Wounds of Peripheral Nerves. By LIEUT.-COLONEL J. MICHELL CLARKE, R.A.M.C.(T.), M.D., F.R.C.P. ... ... ... ... 57

The Prevention of Sepsis in War Wounds, with special reference to the Carrel-Dakin Method. By LIEUT.-COLONEL JAMES SWAIN, R.A.M.C. C.B., M.S., M.D. Lond., F.R.C.S. ... ... ... ... 68

Acute Pancreatitis, with special reference to its treatment, and a record of three cases presenting unusual features. By MAJOR CHARLES A. MORTON, F.R.C.S. ... ... ... ... ... ... 80

Rectal Diverticula as a Causative Factor in Pelvic Inflammation in Women. By MAJOR WALTER C. SWAYNE, M.D. Lond. and Bristol ... ... ... ... ... ... ... ... ... ... 91

Reviews of Books ... ... ... ... ... ... ... ... ... 96

Editorial Notes ... ... ... ... ... ... ... ... ... 107

Obituary Notices ... ... ... ... ... ... ... ... ... 110

